# Consecutive Antibiotic Shortages Highlight Discrepancies between Microbiology and Prescribing Practices for Intra-abdominal Infections

**DOI:** 10.1128/AAC.01980-20

**Published:** 2021-04-19

**Authors:** Stacy C. Park, Grace R. Gillis-Crouch, Heather L. Cox, Lindsay Donohue, Rena Morse, Kasi Vegesana, Amy J. Mathers

**Affiliations:** a Division of Infectious Diseases and International Health, Department of Medicine, University of Virginia Health System, Charlottesville, Virginia, USA; b Department of Pharmacy Services, University of Virginia Health System, Charlottesville, Virginia, USA; c Health Information & Technology, University of Virginia Health System, Charlottesville, Virginia, USA; d Clinical Microbiology Laboratory, Department of Pathology, University of Virginia Health System, Charlottesville, Virginia, USA

**Keywords:** antibiotic shortage, antimicrobial stewardship, piperacillin-tazobactam, cefepime, intra-abdominal infection, antimicrobial prescribing

## Abstract

Piperacillin-tazobactam (TZP) is frequently used for intra-abdominal infection (IAI). Our institution experienced consecutive shortages of TZP and cefepime, providing an opportunity to review prescribing patterns and microbiology for IAI. Hospitalized adult patients treated for IAI, based on prescriber selection of IAI as the indication within the antibiotic order, between March 2014 and February 2018 were identified from the University of Virginia Clinical Data Repository and Infection Prevention and Control Database. Antimicrobial utilization, microbiologic data, and clinical outcomes were compared across four 1-year periods: preshortage, TZP shortage, cefepime shortage, and postshortage. There were 7,668 episodes of antimicrobial prescribing for an indication of IAI during the study period. Cefepime use for IAI increased 190% during the TZP shortage; meanwhile ceftriaxone use increased by only 57%. There was no increase in in-house mortality, colonization with resistant organisms, or Clostridioides difficile infection among patients treated with IAI during the shortage periods. Among a subset of cases randomly selected for review, *Pseudomonas* sp. was a rare cause of IAI, but antipseudomonal antibiotics were commonly prescribed empirically. We observed a large increase in cefepime utilization for IAI during a TZP shortage that was not warranted based on the observed frequency of identification of *Pseudomonas* sp. as the causative organism in IAI, suggesting a need to revisit national guideline recommendations.

## Abstract (precis)

Piperacillin-tazobactam (TZP) is frequently used for intra-abdominal infection (IAI). Our institution experienced consecutive shortages of TZP and cefepime, providing an opportunity to review prescribing patterns and microbiology for IAI.

## INTRODUCTION

Drug shortages, and particularly antibiotic shortages, are an increasingly common problem faced by medical centers worldwide (1, 2). As exact therapeutic equivalents do not usually exist, substitutions made in the setting of shortages may increase use of agents that are less effective, more toxic, or unnecessarily broad in antibacterial spectrum compared to first-line therapy (3–5). A 2016 study of a piperacillin-tazobactam (TZP) shortage showed an 111% increase in meropenem use at one institution (5). The impact of antibiotic shortages on antimicrobial resistance and rates of Clostridioides difficile infection are also concerns. A 2017 study showed a near doubling of the frequency of vancomycin-resistant enterococci (VRE) and carbapenem-resistant *Enterobacterales* during a TZP shortage (6), and a multicenter study of hospitals that experienced TZP shortages showed an increase in hospital-onset C. difficile infection among those that responded by shifting antibiotic usage toward “high-risk” antibiotics (7).

Beginning in March 2015, supplies of TZP at our institution entered a period of shortage lasting approximately 1 year. Members of the stewardship team at our institution noted an apparent surge in cefepime utilization during this period. This was followed almost immediately by a year-long shortage of cefepime. As TZP is commonly prescribed for the indication of intra-abdominal infection (IAI), we sought to characterize changes in antimicrobial prescribing and microbiology in patients with IAI during these shortages, with the hypothesis that *Pseudomonas* sp. is frequently covered empirically and infrequently isolated in IAI. We also assessed whether there were changes in rates of colonization with resistant organisms (methicillin-resistant Staphylococcus aureus [MRSA] and VRE) or C. difficile infection, as this has been noted by others in shortage scenarios (6, 7). Finally, we also examined length of stay, intensive care unit (ICU) transfer, and in-hospital mortality as outcomes that could potentially be affected by disruption of prescribing patterns (and potentially suboptimal substitutions).

## RESULTS

**FIG 1:**
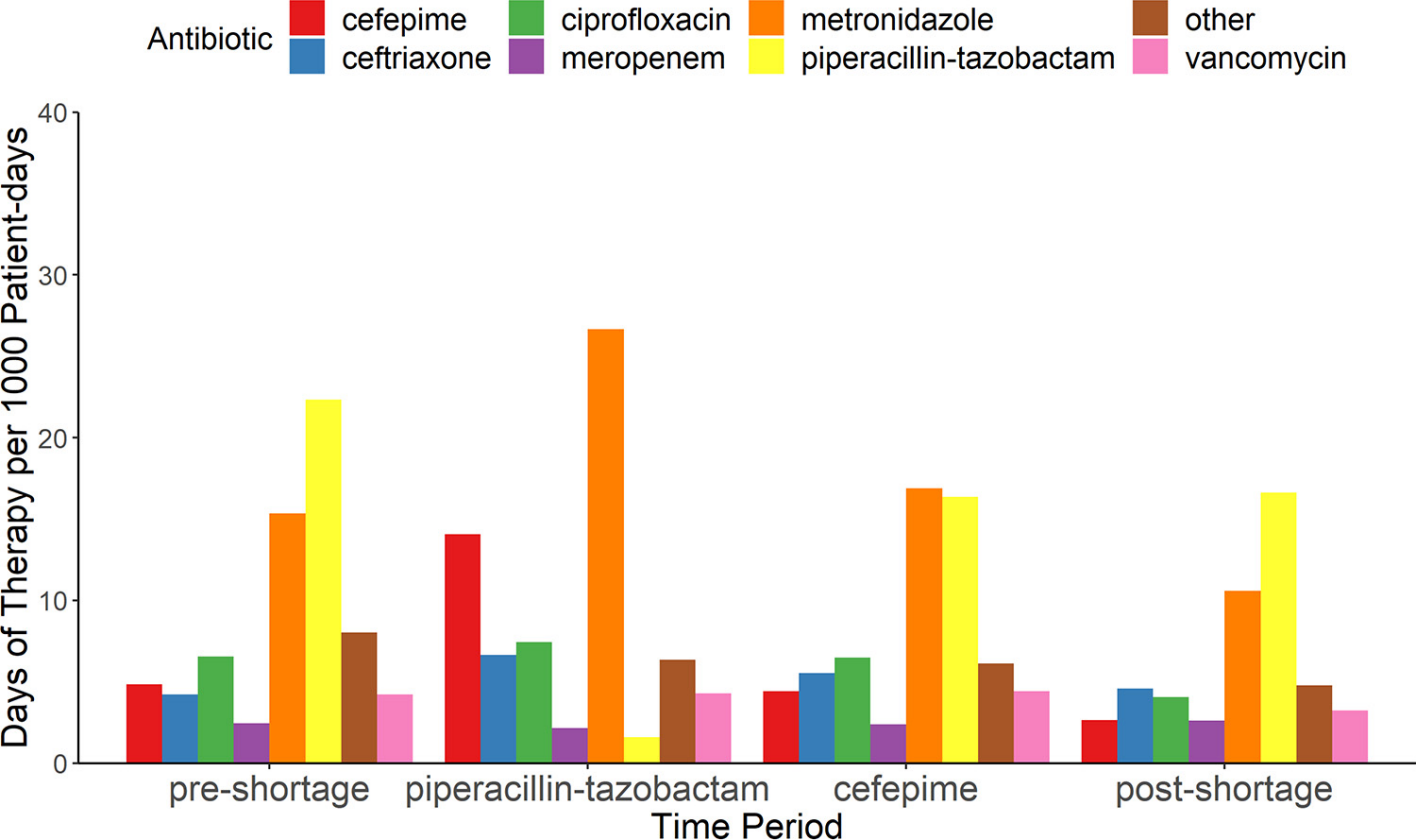
Antimicrobial utilization for IAI before, during, and after consecutive piperacillin-tazobactam and cefepime shortages.

There were 7,668 episodes of antimicrobial prescribing for an indication of IAI across all four time periods (Table 1). During the TZP shortage, there was a 93% reduction in TZP usage for an indication of IAI (measured in days of therapy per 1,000 hospitalized patient-days), a 190% increase in cefepime usage, a 57% increase in ceftriaxone usage, a 13% increase in ciprofloxacin usage, and a 74% increase in metronidazole usage compared to the preshortage period (Fig. 1). During the cefepime shortage, there was a 69% reduction in cefepime usage relative to the preceding (TZP shortage) period; however, there was only a 9% reduction compared to the preshortage period, and cefepime usage was lowest in the postshortage period. Meropenem was a restricted agent requiring antimicrobial stewardship approval throughout the time periods; usage was stable throughout the shortage periods. Vancomycin usage decreased across all four periods: there was a 40% reduction in the postshortage relative to the preshortage period. Rates of VRE colonization declined over time (TZP shortage odds ratio [OR] = 0.73 [95% confidence interval, 0.59 to 0.89], cefepime shortage OR = 0.56 [0.45 to 0.69], postshortage OR = 0.43 [0.33 to 0.54]; the referent is the baseline period), as did MRSA colonization (TZP shortage OR = 0.72 [0.53 to 0.97], cefepime shortage OR = 1.13 [0.87 to 1.48], postshortage OR = 0.31 [0.20 to 0.45]). The number of positive C. difficile PCR tests was similar across all time periods (*P* = 0.20) (Table 2). Length of stay and number of ICU admissions were similar across all time periods (*P* = 0.71 and *P* = 0.21, respectively), and in-house mortality was significantly higher in the baseline period compared to all other periods (TZP shortage OR = 0.77 [0.62 to 0.95], cefepime shortage OR = 0.71 [0.57 to 0.88], postshortage OR = 0.77 [0.62 to 0.95]).

**TABLE 1:** Characteristics across time periods

Period	Total no. of:		Median (IQR)	
	Patients with IAI	Patient-days	Age (yr)	Charlson score
Preshortage	2,107	190,145	58 (47.0–68.5)	2 (0–4)
Piperacillin-tazobactam shortage	1,896	216,707	58 (46.0–69.0)	1 (0–4)
Cefepime shortage	1,888	194,919	59 (45.0–70.0)	0 (0–4)
Postshortage	1,777	198,008	58 (46.0–70.0)	1 (0–4)

**TABLE 2:** Outcomes among inpatients who received antibiotics for IAI by time period

Outcome	Value for period				*P* value^*a*^
	Preshortage	TZP shortage	Cefepime shortage	Postshortage	
Days of therapy per 1,000 hospital patient-days^*b*^
Cefepime	4.85	14.08	4.41	2.66	<0.001
Ceftriaxone	4.23	6.66	5.52	4.57	<0.001
Ciprofloxacin	6.56	7.43	6.49	4.05	<0.001
Meropenem	2.45	2.17	2.38	2.50	0.11
Metronidazole	15.34	26.66	16.90	10.60	<0.001
Piperacillin-tazobactam	22.34	1.60	16.35	16.64	<0.001
Other	12.24	10.65	10.56	8.02	<0.001
Vancomycin	8.03	6.36	6.14	4.79	<0.001

Length of admission (days) [median (IQR)]	5 (2–11)	5 (2–12)	5 (2–11.25)	5 (2–11)	0.71

No. (%)
With ICU admission^*c*^	115 (5.46)	86 (4.54)	105 (5.56)	108 (6.08)	0.21
With in-hospital mortality	222 (10.54)	157 (8.28)	144 (7.63)	136 (7.65)	0.002
VRE positive	258 (12.24)	175 (9.23)	136 (7.20)	100 (5.63)	<0.001
MRSA positive	112 (5.32)	74 (3.90)	113 (5.99)	30 (1.69)	<0.001
C. difficile positive	111 (5.27)	109 (5.75)	84 (4.45)	80 (4.50)	0.20

a Based on Kruskal-Wallis test for continuous variables and chi-square test for categorical variables.

b Total hospitalized patient-days per time period.

c Percent of admitted patients with IAI selected as the indication for an antibiotic.

### Review of selected cases.

Among 416 (∼5%) cases randomly selected for in-depth chart review, categorization of cases and positive C. difficile tests were similar across all time periods (Table 3), with the exception that there were fewer erroneous indication selections in the postshortage period. The proportion of cases with an infectious disease consult doubled in the TZP shortage period relative to the preshortage period and remained stable in subsequent periods, including the postshortage period.

**TABLE 3:** Characteristics of selected cases from different time periods

Characteristic	No. (%) of cases in time period			
	Preshortage (*n* = 108)	TZP shortage (*n* = 100)	Cefepime shortage (*n* = 100)	Postshortage (*n* = 108)
CA-IAI	22 (20.4)	23 (23)	24 (24)	22 (20.4)
HA-IAI	29 (26.9)	27 (27)	33 (33)	36 (33.3)
IAI possible	38 (35.2)	32 (32)	25 (25)	38 (35.2)
Erroneous	19 (17.6)	18 (18)	18 (18)	12 (12)
Infectious diseases consult	10 (9.3)	22 (22)	22 (22)	20 (18.5)
C. difficile positive	3 (2.8)	4 (4)	4 (4)	3 (2.8)
Organism(s) isolated	16 (14.8)	19 (19)	27 (27)	30 (27.8)
Bacteremia	4 (3.7)	5 (5)	7 (7)	10 (9.3)
Empiric antipseudomonal regimen^*a*^
CA-IAI	12 (54.5)	5 (21.7)	11 (45.8)	11 (50)
HA-IAI	20 (70)	14 (51.9)	22 (66.7)	26 (72.2)
Neutropenia	2 (1.9)	4 (4)	2 (2)	3 (2.8)

a Defined as piperacillin-tazobactam, cefepime, or meropenem.

### Microbiology.

Among the 416 cases, 92 (22.1%) had at least one organism isolated that was attributed to an intra-abdominal source. In the preshortage period, fewer cases (16/108; 15%) had an associated organism identified relative to subsequent time periods (Table 3). The most commonly isolated organism was Escherichia coli, followed by Bacteroides fragilis. For community-acquired IAI (CA-IAI) cases, 27/91 (30%) had positive microbiologic data, none of which were *Pseudomonas* sp.; however, 39/91 (43%) reviewed cases of CA-IAI received initial empirical therapy that included antipseudomonal spectrum. For health care-associated IAI (HA-IAI) cases, 58/125 (46%) had positive microbiologic data, 3 of which were Pseudomonas aeruginosa; 82/125 (66%) received initial empirical therapy that included antipseudomonal spectrum. Among cases with positive culture data (*n* = 92), only 3 (3%) were due to P. aeruginosa and 6 organisms (7%) were ceftriaxone-nonsusceptible *Enterobacterales*, and all cases were HA-IAI (Table 4). Cases of IAI due to P. aeruginosa included a patient with recurrent peritoneal dialysis catheter-associated peritonitis (with prior isolation of P. aeruginosa), one with an indwelling biliary drain complicated by cholangitis, and one with inflammatory bowel disease complicated by C. difficile colitis who underwent fecal microbiota transplantation and subsequently developed polymicrobial bacteremia.

**TABLE 4:** Organisms isolated and attributed to IAI among subset of reviewed cases^*a*^

Organism	No. (%) (*n* = 92)	Resistance of note
Facultative and aerobic gram-negative
Escherichia coli	18 (19.6)
*Klebsiella* sp.	11 (12)
*Enterobacter* sp.	10 (10.7)	2 ceftriaxone-nonsusceptible strains
Pseudomonas aeruginosa	3 (3.3)
*Raoultella* sp.	4 (4.3)	2 ceftriaxone-nonsusceptible strains
*Aeromonas* sp.	3 (3.3)
Citrobacter freundii	2 (2.2)	2 ceftriaxone-nonsusceptible strains
Serratia marcescens	2 (2.2)
Morganella morganii	1 (1.1)
*Moraxella* sp.	1 (1.1)
Kluyvera intermedia	1 (1.1)
Stenotrophomonas maltophilia	1 (1.1)
*Pantoea* sp.	1 (1.1)
Anaerobic bacteria		
Bacteroides fragilis	13 (13.8)
*Lactobacillus* sp.	1 (1.1)
*Prevotella* sp.	2 (2.2)
*Clostridium* sp.	1 (1.1)
Leuconostoc mesenteroides	1 (1.1)
Gram-positive aerobic cocci		
Enterococcus faecium	10 (10.9)	5 VRE strains
Enterococcus faecalis	3 (3.3)
*Streptococcus* sp.	6 (6.5)
Staphylococcus aureus	4 (4.3)	2 MRSA strains
Rothia mucilaginosa	1 (1.1)
Fungi		
Candida albicans	6 (6.5)
Candida glabrata	8 (8.7)
Candida dubliniensis	2 (2.2)
Candida guilliermondii	2 (2.2)
Candida lusitaniae	1 (1.1)

a Includes only organisms identified that were attributed to an intra-abdominal source (i.e., microbiology for erroneous selections was not included). More than 1 organism was isolated in 20 cases. Mixed flora was isolated in 26 cases.

*Enterococcus* sp. was isolated in 13 cases, 11 of which were HA-IAI. One community-acquired case was a patient with metastatic gallbladder carcinoma admitted for cholangitis, and the other was a patient on peritoneal dialysis who presented with septic shock due to VRE bacteremia, potentially due to peritonitis versus endocarditis. Staphylococcus aureus was rarely identified as the causative organism (*n* = 4, 2 of which were MRSA) and was found exclusively in patients following procedures (3 patients had had abdominal surgery and 1 had had endoscopic retrograde cholangiopancreatography with common bile duct stent placement).

## DISCUSSION

TZP is commonly prescribed for IAI in hospitalized patients and has antipseudomonal activity as well as providing coverage of increasingly resistant E. coli; however, the actual antimicrobial coverage intent may not be well understood by all prescribers (8). The microbiologic data from the subset of cases we reviewed demonstrate the relative rarity of *Pseudomonas* sp. as the causative organism, even for hospital-associated cases, in IAI. P. aeruginosa is infrequently carried in the gut of healthy humans and thus would not be generally expected to play a large role in IAI, especially from the community (9, 10). Even in ICU patients without specific perturbations in their gut flora, P. aeruginosa was infrequently identified compared to *Enterobacterales* (11). Here, ceftriaxone-nonsusceptible *Enterobacterales* were more common than *Pseudomonas* sp., and all had elevated MICs of cefepime, TZP, or both. Despite this, antipseudomonal antibiotics were commonly used for IAI, and the increased cefepime usage in the setting of a TZP shortage particularly highlights the discrepancy between prescribing practices and the microbiology of IAI.

The most recent Infectious Diseases Society of America (IDSA) guidelines for IAI recommend that empirical therapy for health care-associated IAI (HA-IAI) be driven by local microbiologic results, but they also state, “to achieve empirical coverage of likely pathogens, multidrug regimens that include agents with expanded spectra of activity against Gram-negative aerobic and facultative bacilli may be needed. These agents include meropenem, imipenem-cilastatin, doripenem, piperacillin-tazobactam, ceftazidime, or cefepime plus metronidazole…” (12). The empirical use of antimicrobial regimens with broad-spectrum Gram-negative organisms is also recommended for CA-IAI (12). Revised 2017 guidelines provided by the Surgical Infection Society highlight the need for individual risk assessment for various resistant pathogens; however, they also recommend the use of antimicrobials with broad-spectrum coverage of Gram-negative organisms for “high-risk” patients with CA-IAI and patients with HA-IAI (13). Other guidelines focus more on severity of illness as the indication for the empirical use of agents with broader spectra (14, 15). However, our data suggest that considering all patients in these groups as being at high risk for IAI due to resistant Gram-negative organisms results in significant overutilization of agents with expanded spectra for Gram-negative organisms, and this recommendation may need to be revisited in the next edition. Furthermore, when resistant Gram-negative organisms were isolated from an abdominal source, extended-spectrum beta-lactamase (ESBL)-producing *Enterobacterales* were more common than P. aeruginosa; thus, a carbapenem may be the superior agent over antipseudomonal beta-lactams, such as cefepime, when more broad-spectrum Gram-negative coverage is deemed appropriate (16, 17). This highlights the importance of institutional guidelines and the need for better ways to identify the minority of patients with IAI due to resistant Gram-negative organisms.

Existing guidelines additionally recommend considering empirical antimicrobial coverage directed against MRSA for patients with HA-IAI “who are known to be colonized with the organism or who are at risk of having an infection due to this organism because of prior treatment failure and significant antibiotic exposure” (12). Among the cases reviewed in our study, Staphylococcus aureus was rarely isolated and was exclusively found in patients with a history of recent surgery or procedure. Recommendations for empirical coverage of *Enterococcus* spp. generally include consideration based on individual patient risk given clinical characteristics and/or severity of illness, though risk factors are variably and vaguely defined (12–15). Interestingly, the marked increase in cefepime use during the TZP shortage in this study was not accompanied by a concomitant surge in vancomycin use, suggesting that coverage of *Enterococcus* spp. may not play a large role in prescribers’ antibiotic decision-making for IAI at our institution.

The antimicrobial prescribing data from this study also highlight important points pertinent to antibiotic stewardship efforts in the setting of shortages. We observed a far more dramatic decrease in utilization of the shortage antibiotic during the TZP shortage. This may be due in part to the greater severity of the TZP shortage at our institution relative to the cefepime shortage; however, it is also likely that the restriction and preauthorization approach had a greater impact on prescribing practices than the prospective audit and feedback approach (18). Since the challenge in antibiotic shortage situations is helping prescribers to choose the most appropriate alternative antibiotic (not reducing utilization of a target antibiotic), strategies that maximize interaction with stewardship teams or otherwise provide more tailored guidance may be more advisable (19). At our institution, prescribers seemingly learned that there was a shortage of TZP and preferentially chose another antibiotic rather than calling the antibiotic stewardship team for prior authorization, which is known to be a potentially frustrating process for clinicians (20). This likely contributed to the large surge in cefepime utilization, whereas discussion with the antibiotic stewardship team might have led to more ceftriaxone use, consistent with the institutional guidance.

Our study was not designed to evaluate the reasons that prescribing practices were inconsistent with the provided guidance; however, possible explanations include low read rates for the email that was circulated or selective retention of information from the email (of note, recommendations for nosocomial infections were listed first, and the recommendations for CA-IAI were listed last). We also recognize that passive information has not been shown to frequently change practice (21). Other possible contributing factors include limited understanding of antimicrobial spectrum or misconceptions about the frequency of *Pseudomonas* sp. involvement in IAI (22, 23) and the availability of different sets of guidelines that prescribers may reference (12–15).

Our study was designed primarily to look at changes in antibiotic prescribing practices for IAI during specific β-lactam antibiotic shortages. Thus, our ability to detect overall changes in colonization rates of resistant organisms and rates of C. difficile, which have been previously associated with shortages (6, 7), was limited, and we cannot rule out contributions of temporal factors to the observed colonization rates. However, there was no increase in resistant-organism colonization or C. difficile infection among patients with IAI (as identified by selection of IAI as the indication for antibiotic orders). There also was no increase in in-hospital mortality during shortage periods. In fact, in-hospital mortality was highest in the preshortage period and declined over time. Based on the reviewed subset of cases, the frequency of infectious disease consultation and identification of pathogens was lowest in the preshortage period. However, the observational nature of this study precludes making conclusions about causality.

Another limitation of this study is the inclusion of patients based on a prescriber selecting IAI as the indication for an antibiotic order. Per our review of a subset of cases, approximately 12% of these indications were erroneous selections. However, the majority of erroneous selections were for patients receiving prophylaxis for spontaneous bacterial peritonitis in the setting of variceal bleeding (typically with ceftriaxone) or perioperative prophylaxis for patients undergoing abdominal surgery, both instances in which prescribers would be targeting similar organisms and which would be more likely to inflate ceftriaxone usage. Inclusion based on this parameter allowed the large number of patients, which is a strength, and allowed assessment at the point of prescribing for IAI, which is highly relevant for analysis of prescribing behavior from a stewardship standpoint. Furthermore, the erroneous selections did not impact the analysis of the microbiologic etiologies or rates of empirical use of antipseudomonal agents, as these were analyzed based on classification within the reviewed subset of cases. However, this method of identification for inclusion likely did miss some cases of IAI wherein IAI was not selected as the indication by prescribers at the time of the order.

Antibiotic shortages are a barrier to best antimicrobial stewardship practices (5, 24). We found that the alternative selections prescribers made in the setting of TZP shortage were suboptimal, with cefepime being substituted at a rate that was not supported by the microbiologic epidemiology of IAI at our institution. Future research and guideline updates should seek to refine indications and recommendations for various resistant Gram-negative organisms (e.g., *Pseudomonas* sp. and ESBL-producing *Enterobacterales*). Institutional guidelines may be critical, particularly in the setting of antibiotic shortages; however, the best method for optimizing adherence to such guidance remains unclear.

## MATERIALS AND METHODS

### Data.

Antimicrobial usage, infection rate and culture data, and patient demographics were extracted from the University of Virginia Clinical Data Repository and Infection Prevention and Control Database. Adult patients admitted to the University of Virginia Health System in Charlottesville, VA, between March 2014 and February 2018 who received an antimicrobial with “intra-abdominal infection” as the indication in the electronic medical record (Epic, Verona, WI) during their admission were included. Indication selection was required to sign intravenous antibiotic orders throughout the study period; prescribers could select one of 20 provided indications or enter a free-text indication. These patients were placed in one of four 1-year-long time periods: preshortage (March 2014 to February 2015), TZP shortage (March 2015 to February 2016), cefepime shortage (March 2016 to February 2017), or postshortage (March 2017 to February 2018). Patients less than 18 years of age and duplicate patients readmitted within 30 days of the initial admission were excluded. Antimicrobial utilization was assessed using days of therapy (DOT) per 1,000 patient-days. Antimicrobials on the inpatient hospital formulary that are commonly prescribed for IAI at our institution were specifically measured, including TZP, cefepime, ceftriaxone, ciprofloxacin, metronidazole, meropenem, and vancomycin. Usage of other antimicrobials for IAI was also measured in one composite category. Positivity for VRE or MRSA on surveillance screening and positive C. difficile PCR (GeneXpert; Cepheid, Sunnyvale, CA) were also assessed during these four periods, as well as length of admission, admission to an ICU, and in-hospital mortality. There were no significant changes to infection control practice regarding contact precautions or surveillance screening criteria for MRSA or VRE during the study period. In 2017, a previously described diagnostic stewardship initiative including a computerized clinical decision support tool for C. difficile testing was introduced (25), which was associated with reductions in overall testing but not a change in the percentage of positive tests across all hospitalized patients.

### Institutional antimicrobial stewardship practices.

**FIG 2:**
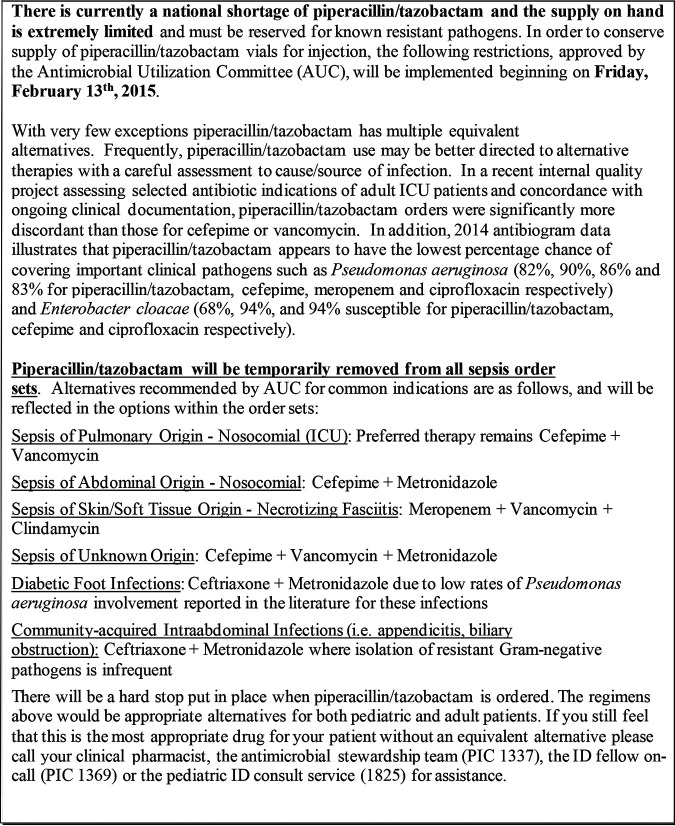
Guidance provided via email in the setting of the TZP shortage.

During the TZP shortage, a 24/7 formulary restriction and preauthorization strategy was used, with a physician leader primarily holding the pager during that time, while during the cefepime shortage, a prospective audit with feedback approach was used and was largely led by an infectious diseases-trained pharmacist. Guidance regarding preferred substitutions for various indications, including community-acquired and nosocomial IAI, was distributed via email during the TZP shortage. Cefepime plus metronidazole was recommended for nosocomial sepsis of abdominal origin, and ceftriaxone plus metronidazole was recommended for community-acquired IAI (CA-IAI) (Fig. 2). Guidance was not provided during the cefepime shortage for IAI, as cefepime was not considered a typical first-line choice for this infection. Meropenem was a restricted agent requiring prior authorization by the antimicrobial stewardship team throughout all time periods.

### In-depth review of cases.

A subset of cases (approximately 5%) were electronically randomly selected for in-depth chart review. Each case was categorized as CA-IAI, HA-IAI, possible IAI, or erroneous indication selection. HA-IAI was defined using the Surgical Infection Society Guidelines on IAI (13), with the exception that use of broad-spectrum antimicrobial therapy during the preceding 90 days was defined as intravenous antimicrobial exposure. Possible IAI included cases in which IAI was one of multiple possible diagnoses and in which there was potential for IAI (e.g., antibiotics were administered in the setting of esophageal perforation; however, a clinically evident infection did not subsequently develop). Cases in which the prescriber inappropriately chose IAI as the indication were categorized as erroneous (e.g., for peri-operative prophylaxis after abdominal surgery or prophylaxis for spontaneous bacterial peritonitis in patients with cirrhosis and variceal bleeding). Additional information extracted from the chart during in-depth review included the initial antibiotic chosen, culture data, C. difficile testing data, infectious diseases consult presence, and narrative summary of the hospital course. Culture results considered attributable to IAI included blood cultures in the setting of clinically diagnosed IAI (excluding those consistent with blood culture contamination) or culture specimens obtained via surgical or percutaneous drainage (e.g., drained abscesses).

### Analysis.

Data analysis was performed with R using the Stats package (R, version 3.5.1). The Kruskal-Wallis test was used for continuous variables, and a chi-square test was performed for categorical variables. Antimicrobial usage data were reported as DOT per 1,000 hospitalized patient-days for each period. For binary outcomes, logistic regression was performed to compare rates across time periods when the chi-square test indicated a significant difference between time periods. Descriptive statistics were used for analysis of data from the in-depth review of a subset of cases.

### Ethics statement.

Database and chart review were approved by the University of Virginia Institutional Review Board (IRB no. 18393 and 20562) with a waiver of consent.
